# Estrogen: An Emerging Regulator of Insulin Action and Mitochondrial Function

**DOI:** 10.1155/2015/916585

**Published:** 2015-03-26

**Authors:** Anisha A. Gupte, Henry J. Pownall, Dale J. Hamilton

**Affiliations:** ^1^Bioenergetics Laboratory, Houston Methodist Research Institute, Weill Cornell Medical College, 6565 Fannin Street, Houston, TX 77030, USA; ^2^Atherosclerosis & Lipoprotein Research, Methodist DeBakey Heart and Vascular Institute, Houston Methodist Research Institute, Weill Cornell Medical College, 6565 Fannin Street, Houston, TX 77030, USA; ^3^Houston Methodist Department of Medicine, Weill Cornell Medical College, 6550 Fannin, Suite 1001, Houston, TX 77030, USA

## Abstract

Clinical trials and animal studies have revealed that loss of circulating estrogen induces rapid changes in whole body metabolism, fat distribution, and insulin action. The metabolic effects of estrogen are mediated primarily by its receptor, estrogen receptor-*α*; however, the detailed understanding of its mechanisms is incomplete. Recent investigations suggest that estrogen receptor-*α* elicits the metabolic effects of estrogen by genomic, nongenomic, and mitochondrial mechanisms that regulate insulin signaling, substrate oxidation, and energetics. This paper reviews clinical and experimental studies on the mechanisms of estrogen and the current state of knowledge regarding physiological and pathobiological influences of estrogen on metabolism.

## 1. Introduction

Estrogens are important participants in metabolic regulation. Loss of the main circulating estrogen, 17*β*-estradiol (E2), due to either natural or surgical menopause has effects that go beyond reproductive health. E2-deficiency and impairment of its cellular action lead to an abrupt reduction in metabolic rate, shift to increased central adiposity, dyslipidemia, and progression of metabolic syndrome (MetS). Together these changes increase the risk of nonalcoholic steatohepatitis, type 2 diabetes, and cardiovascular disease and its complications [1]. With increasing life expectancies, women now spend three to five decades of their life in E2-deficiency and experience health challenges from which E2 had previously provided protection. However, postmenopausal replacement of E2 has been controversial, primarily because of the risk of oncogenicity and the adverse outcomes on cardiovascular disease (CVD) seen in the Women's Health Initiative (WHI) trials [2]. Yet women who take hormone replacement therapy (HRT) seem to enjoy the metabolic benefits of E2; they are more energetic, have better glucose metabolism, do not have hot flashes, can better control their weight, and benefit from improved bone density, all to the extent that they decide that these benefits outweigh the risks [3]. The mechanisms by which E2 regulates metabolism and glucose homeostasis are not well understood. A deeper understanding of mechanisms underlying E2 metabolism might better inform decisions on the design of E2 receptor modulators that would optimize metabolic benefits for disease prevention and treatment without the associated reproductive, oncogenic, or CVD risks. There is a growing awareness of the role of E2 in metabolism via its regulation of mitochondrial function. This review comprehensively presents and discusses the mechanisms by which E2 regulates mitochondrial function and insulin action.

## 2. Clinical Studies Document Increased Risk of MetS and Diabetes after Loss of E2

Large clinical studies have revealed a robust protective role of E2 against MetS and diabetes. In a population-based prospective cohort study, diabetes risk was reduced by 62% in women with current HRT use compared with individuals who never used HRT [4]. Similar other large-scale trials have shown benefits of HRT on diabetes in postmenopausal women [5, 6]. Weight gain, with its associated predisposition to diabetes, commonly occurs with menopause and is primarily attributable to aging. However, beyond the weight gain itself, changes in body composition that are classically associated with insulin resistance, such as increase in visceral adiposity, have been independently linked to the menopausal transition [7]. Despite the relationship between menopause and weight/body composition changes, a randomized double-blind, placebo-controlled trial of E2+progestin replacement in women with coronary heart disease reported stabilization of fasting glucose levels and 35% reduction in incidence of diabetes with no changes in weight and waist circumference [5]. HRT also improved glucose control in women with preexisting diabetes [7], and E2 given in a moderate dose (0.625 mg) increased insulin sensitivity; however, higher doses (1.25 mg) or progestins cotreatment attenuated this benefit [8]. Taken together, clinical studies have confirmed the protective effects of E2 on MetS and diabetes, but these studies need to be followed by studies in animal models to identify mechanisms underlying the above discrepancies and patient-group selective effects.

## 3. Biochemical Mechanisms of E2 Signaling

**Figure 1:**
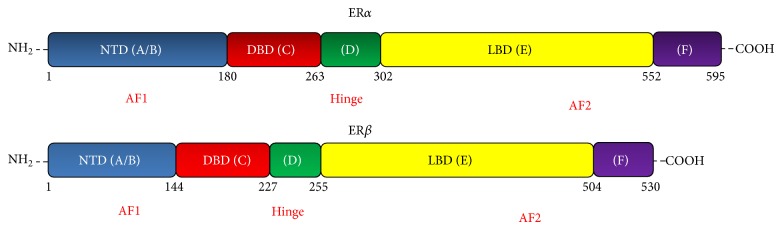
Domain structure of ER*α* and ER*β*. NTD: N-terminal domain, DBD: DNA-binding domain, and LBD: ligand-binding domain.

E2 mediates its effects via 3 receptors—E2 receptor *α* (ER*α*), E2 receptor *β* (ER*β*), and the newly described G protein-coupled E2 receptor 1 (GPER). Variations in the action of E2 depend upon the relative distribution and abundance of the ERs across different tissues and within intracellular locations. ER*α* is the primary ER in most reproductive tissues as well as insulin-sensitive tissues. The ERs have structural similarities with other members of the nuclear receptor family [9]. The N-terminal A/B domain contains an activation function1 (AF1), which is ligand-independent and has promoter- and cell-specific activity. The DNA-binding domain resides in the C-domain whereas the nuclear localization signal is in the D-domain. The C-terminal E-domain is the ligand-binding domain, which contains a ligand-dependent AF2. The function of the F-domain remains undefined (see Figure 1) [9].

**Figure 2:**
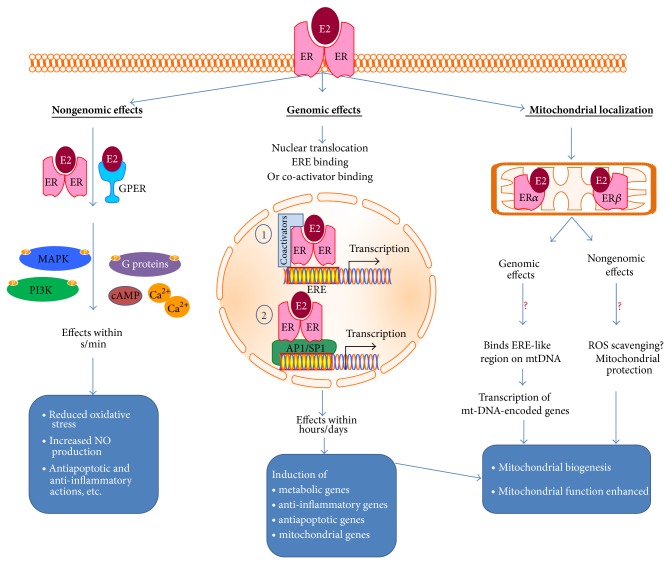
Mechanisms of estrogen action: binding and dimerization of ERs by E2 trigger nongenomic, genomic, and mitochondrial effects. Nongenomic effects may be mediated by E2-ER or by E2 bound to GPER by activating signaling molecules like MAPK, PI3K, G-proteins, and more to elicit immediate actions. Genomic effects are mediated by nuclear translocation of E2-ER complex and either (1) direct binding with estrogen response elements (ERE) along with coactivators to form a transcription complex or (2) binding to transcriptional coactivators to induce gene transcription indirectly. ERs may also localize to mitochondria to induce potentially genomic and nongenomic actions, the mechanisms of which are not well understood.

In the classical E2 signaling pathway, two ERs dimerize when stimulated by E2 binding and then translocate to the nucleus, bind to E2 response elements (ERE), and elicit a transcriptional response (see Figure 2). The nonclassical E2 signaling pathway operates independently of ER-ERE binding and involves protein-protein interactions that elicit genomic and nongenomic effects. For instance, the ERs may cross talk with the transcription factors AP1 and SP1 to indirectly regulate transcription. Although ER*α* and ER*β* have DNA- and ligand-binding domain homology, they differ especially in their N- and C-terminal sequences. There is some evidence that ER*β* may have less nuclear transcriptional activity than ER*α* [10]. The nongenomic effects involve interaction of the membrane-localized ER with adaptor proteins such as c-Src and downstream rapid signaling via mitogen-activated protein kinase (MAPK), G-proteins, protein kinase B (PKB)/PI3K, and protein kinase C (PKC). Moreover, E2 also signals nongenomically via GPER. This signaling is rapid and triggers the release of intracellular Ca^+2^, cAMP production, or c-Src activation with subsequent activation of MAPK or calcium calmodulin-dependent kinases [11, 12]. The extent to which E2 regulates energy homeostasis via these nonclassical ER signaling pathways remains unclear. Using gene knock-in mice that express mutant (E207A/G208A) ER*α* that can only signal through the noncanonical pathway, Park et al. found that nonclassical ER*α* signaling mediates the major effects of E2 on energy balance [13].

The activity of estrogen also depends on its bioavailability which is primarily determined by the sex hormone-binding globulin (SHBG). SHBG transports and regulates activities of androgens and estrogen by regulating plasma distribution and access of these hormones to their target tissues [14]. However, several single nucleotide polymorphisms (SNP) have been described in SHBG, some of which are associated with the MetS. For instance, a common SNP (rs6259) retards the plasma clearance of SHBG and is negatively associated with type 2 diabetes [15].

## 4. Relationship of E2 and Insulin

After menopause most women face a dramatic increase in central obesity, insulin resistance, and dyslipidemia, all factors associated with the MetS [16]. Likewise ER*α* knockout mice are obese and insulin resistant and have decreased energy expenditure, decreased locomotion, abnormal glucose homeostasis, hyperleptinemia, and hyperinsulinemia [17–19]. ER*α* activation with specific agonists reverses high fat diet- (HFD-) induced insulin resistance [20], whereas ER*β* knockout mice display improved insulin sensitivity and glucose tolerance [21], suggesting that ER*α* plays a primary role in insulin-glucose homeostasis. These findings are consistent with human studies in which estrogen-deficient men and women with Cyp19 aromatase deficiency and a male patient with ER*α* deficiency exhibited insulin resistance, impaired glucose metabolism, and hyperinsulinemia [22]. E2 treatment reversed the insulin resistance only in the aromatase deficient patients [23].

E2 may regulate insulin action directly via actions on insulin-sensitive tissues or indirectly by regulating factors like oxidative stress, which contribute to insulin resistance. In skeletal muscle, ER*α* is thought to have a positive effect on insulin signaling and GLUT4 expression whereas ER*β* may be prodiabetogenic and cause reduced GLUT4 expression [24, 25]. Our group showed altered ER*α* expression primarily in the adipose tissue of ovariectomized (OVX) mice treated with HFD [26]. But* in vivo* stimulation of ER*α* with its agonist PPT increased insulin-stimulated glucose uptake in slow- and fast-twitch skeletal muscles along with activation of signaling intermediates whereas activation of ER*β* with DPN did not alter insulin action [27]. The role of ERs in liver has been studied in liver-specific ER*α* knockout mice fed a HFD. These mice have decreased insulin sensitivity during a hyperinsulinemic euglycemic clamp and insulin failed to suppress endogenous glucose production, indicative of hepatic insulin resistance [28]. Hepatic lipotoxicity and impaired gluconeogenesis have been described in OVX mice and one study indicated that changes in gluconeogenesis may be unrelated to E2-deficiency in OVX mice [29]. E2 may also mediate its protective effects on insulin action via reduction of inflammation [30]. Hematopoietic or myeloid-specific ER*α* exerts important effects on global insulin action and MetS [31]. Effects of E2 in metabolism are also centrally controlled at the level of the hypothalamus regulating appetite [32], and thus obesity due to increased appetite in E2-deficiency contributes to reduced insulin sensitivity. Yonezawa et al. compared HFD-OVX mice receiving subcutaneous versus intracerebroventricular E2 to delineate the contribution of central versus peripheral effects of E2 on metabolism and insulin action [33]. While both treatments improved insulin sensitivity, the authors found that subcutaneous E2 decreased expression of TNF*α*, lipoprotein lipase, and fatty acid synthase, whereas intracerebroventricular E2 upregulated energy expenditure via activation of brown adipose tissue thermogenesis and suppression of hepatic gluconeogenesis. E2 also regulates pancreatic *β* cell function likely through an ER*α* mechanism. ER*α* knockout mice have increased susceptibility to oxidative stress, precipitating beta cell apoptosis and insulin-deficient diabetes [34]. The protective effects of E2 on *β* cells are primarily nongenomic and likely independent of ERs since 17*α*-estradiol also mimicked these effects [35]. Taken together, E2 influences glucose homeostasis through multiple organ systems with organ-specific effects acting primarily via ER*α*.

One developing theory of insulin resistance is that chronic oxidative stress activates kinases such as JNK and IKK*β*, which inhibit activation of the insulin signaling intermediates [36, 37]. E2 suppresses oxidative stress likely via both nongenomic and genomic actions [38], by activating pathways that prevent generation of reactive oxygen species (ROS) and increasing efficient scavenging of ROS. It is also likely that some of the effects of loss of E2 on insulin action are due to the increased adiposity associated with E2-deficiency. Both ER*α*- and GPER-deficient mice have increased adiposity and insulin resistance [39].

Treatment with physiological levels of E2 restores insulin sensitivity and glucose tolerance in HFD-fed OVX mice, an effect that was abolished in ER*α*-deficient mice [40]. In ob/ob mice, systemic treatment with the ER*α*-selective ligand PPT improved glucose tolerance and insulin sensitivity [20], reiterating the role of ER*α* in glucose homeostasis. However, only early onset E2 treatment rescued the ovariectomy-induced oxidative stress, reduced brain glucose uptake, and decreased GLUT1 and 3 expression and metabolomics profile changes [38]. Indeed, one interpretation of the results from WHI trials is that HRT was ineffective or possibly detrimental to women when it was started in established postmenopausal women whereas it was beneficial to newly menopausal women [41]. A likely explanation of this observation is that the ratio of ER*α*/ER*β* changes over time with ovariectomy, altering the effect of delayed E2 treatment. Alternatively, the detrimental effects of ovariectomy and possibly from other factors like diet and age are so extensive that delayed E2 treatment has minimal effects. Hence, E2 treatment may more likely prevent rather than reverse preexisting damage.

It seems clear that E2 exerts positive regulation on insulin action. However, this relationship is not reconciled under conditions of high E2 levels such as polycystic ovarian disease, obesity, or pregnancy—all characterized by insulin resistance. Obese postmenopausal women have higher serum E2 levels than lean postmenopausal women [42]. Indeed, supraphysiological doses of estradiol suppressed basal and insulin-stimulated glucose oxidation in human myocytes, whereas low concentrations of E2 increased glucose uptake [43]. This paradoxical relationship begs to question “Is there a difference in the ovary-derived circulating E2 and extragonadal-derived E2, which is thought to have paracrine function (reviewed in [44]) in adipose, breast, brain, muscle, and bone tissue?” Also, why does the adipose tissue in particular secrete E2 and what is the trigger? It is likely that inflammation in obesity induces expression of aromatase, which increases E2 production to suppress the inflammation in a paracrine manner. Moreover, the protective effects of E2 that have been consistently observed in clinical studies against cardiometabolic risks are absent in women with type 1 diabetes even though they have normal E2 levels [45, 46]. Perhaps, E2 treatment for metabolic disorders should be targeted toward those with insulin resistance and MetS with personalized consideration to dose and comorbidities.

Women tend to accrue fat primarily in the subcutaneous regions whereas men tend to have visceral adiposity which is positively correlated with risk for CVD and MetS. After menopause, adiposity shifts from subcutaneous to the visceral area, and subsequently the incidence of CVD and MetS in women increases. Two main mechanisms have been suggested to explain the shift in fat distribution with menopause. (1) Influence of E2 on adrenergic receptors alters the lipid storage characteristics of the fat depots. E2 can shift the balance between lipolytic *β*1-2 receptors and antilipolytic *α*2 adrenergic receptors between the subcutaneous and visceral depots [47]. (2) Altered distribution of ER*α* and ER*β* in adipose depots allows E2 to modulate distribution of fat between the depots. Males have lower ER*α* in their visceral depots and are therefore primed to store more fat viscerally [48]. Mice with a global deficiency of ER*α* have primarily visceral adiposity. After ovariectomy, E2 can reverse visceral adiposity in wild type and ER*β* knockout mice, but not in ER*α* mice, suggesting that the lipolytic effect of E2 is primarily mediated by ER*α*. Also, mice with adipocyte-specific deletion of ER*α* have increased adiposity specifically in the visceral depot [49]. Thus a higher ER*α*/ER*β* ratio in the visceral depot may limit the accumulation of fat in premenopausal women. Another theory suggests that, after menopause, the adipose tissue becomes the primary source of E2, and it is likely that the process of conversion of E2-precursors to E2 by aromatase may occur mainly in the visceral depot [48]. This depot may thus increase in an effort to replenish at least some of the E2-deficiency in menopause.

If E2 is so crucial to metabolism, does it have any significance in males? The male hormone testosterone may regulate much of the metabolism in males, but HFD-fed liver-specific ER*α* knockout male mice have greater impairment of hepatic insulin sensitivity and increased liver triglycerides and diacylglycerides than the wild type floxed controls [28]. Further, the E2-testosterone balance may be crucial in metabolic regulation since progressive testosterone predominance, particularly bioavailable testosterone (ratio of testosterone to sex hormone-binding globulin) in women without HRT or preexisting diabetes and MetS, was independently associated with increased visceral fat and risk of MetS after menopause [50, 51].

## 5. E2 Is an Important Regulator of Mitochondrial Function

Menopausal women often suffer from low energy levels, muscle weakness, tiredness, reduced exercise capacity, and susceptibility to weight gain. Many of these symptoms may result from energy depletion due to mitochondrial dysfunction. Recent molecular studies have shown that E2 plays a regulatory role in mitochondrial function (Figure 2). E2 appears to modulate various aspects of mitochondrial function, including ATP production, generation of mitochondrial membrane potential, mitochondrial biogenesis, and regulation of calcium concentrations [52, 53]; however understanding the mechanisms underlying these mitochondrial effects, especially in humans, is incomplete. ERs might regulate mitochondrial function through either the classical genomic pathway or nongenomic mechanisms. Recent evidence also suggests that ERs may be localized to the mitochondria and elicit their effects directly. ER*α* is essential for most of the E2-mediated increase in mitochondrial respiratory chain (MRC) proteins and antioxidant proteins involved in defense against oxidative stress [54, 55]. ER*β*, however, can downregulate the mRNA expression of nuclear-encoded subunits of the MRC complexes in the vasculature [54]. E2 may also influence mitochondrial function by altering mitochondrial ROS formation [56] and is thought to induce antioxidant responses [57]. Stimulatory effects of E2 or ER activators have been seen on mitochondrial biogenesis regulators, Nrf1/2, TFAM, and PGC1*α* [58]. Indeed, PGC1*α* is required for ER*β*'s cardioprotective effects following trauma-hemorrhage, reiterating the mechanism of ER action via mitochondrial biogenesis [59].

Rats with normal estrous cycles have enhanced mitochondrial respiration compared with OVX rats [60], and, in MCF-7 cells, mitochondrial oxygen consumption was increased 4–6 days after E2 treatment, following increased expression of the MRC components [58]. E2 might have little effect on mitochondrial ATP production under basal conditions; however, the effect may be robust in stressed conditions such as ischemia, toxins, oxidative stress, or HF [52]. Further, aging in combination with E2-deficiency exacerbates mitochondrial dysfunction in menopausal women. Quantitative proteomic analyses identified reduction in mitochondrial proteins primarily associated with MRC complexes which was unique to aged-OVX hearts [61].

The mechanism for the genomic action of E2 on mitochondrial function appears to occur via transcription by nuclear translocation of dimerized, E2-bound ERs or via transcriptional activation of mitochondrial genes by ERs localized within the mitochondria [62]. There is controversy regarding the presence of ERs in the mitochondria, but the consensus is that both ERs reside in the mitochondria at least transitionally [63–65]. The precise function of mitochondrial ERs and the stimuli that induce mitochondrial translocation are not clear. The ERs may elicit transcription via binding to an ERE-like element in the mitochondrial genome. Using electrophoresis mobility assays, Chen et al. [66] reported that ER-containing mitochondrial extracts bound to putative mitochondrial EREs (mtEREs) such that the binding was enhanced with E2 and absent in ER*β*-deficient cells. They also showed that the mtERE-bound mitochondrial protein from the mitochondrial extracts is ER*α* and not ER*β*. These data suggest that mitochondrial ER*α* may interact with mtEREs to directly induce E2-dependent transcription. Another insight into the potential mechanism came from the work of Sanchez et al. [67] who showed that E2 stimulated the relocation of ER*α* to mitochondria where it interacts with hydroxysteroid (17-*β*) dehydrogenase 10 (HSD17B10 or HSD10), a multifunctional protein involved in steroid metabolism that is also a core subunit of the mitochondrial RNaseP complex responsible for the cleavage of mitochondrial polycistronic transcripts. This interaction results in processing of mitochondrial transcripts such that mature RNAs are available for translation. But HSD10 also inactivates E2 to a weaker form, estrone; thus the significance of this interaction requires further investigation.

Nuclear genomic regulation of mitochondrial gene expression by E2 is thought to be mediated by ER-ERE-mediated activation of the transcription factor Nrf1 [58], which in turn activates transcription of nuclear-encoded genes such as mitochondrial transcriptional factor A (TFAM) which regulate the mitochondrial genome. Knockdown of Nrf1 blocked E2-induced mitochondrial biogenesis as well as activity. Nrf1 has an ERE in its promoter region, which binds both ER*α* and ER*β in vitro*. However, small interfering RNA to both ERs revealed that ER*α* mediates the E2-induced transcription of Nrf1 [58]. New evidence suggests that GPER also regulates mitochondrial function by preventing opening of the mitochondrial permeability transition pore, mediated by a nongenomic mechanism via Erk activation [68]. Activation of GPER with its agonist, G1, protects the heart against ischemia reperfusion-injury by protecting the mitochondrial function. Further research is warranted to understand the mitochondrial mechanism of E2 in detail, but its role in mitochondrial dynamics is undeniable.

Impaired mitochondrial function in conditions of impaired E2 signaling may be responsible, at least in part, for insulin resistance. Mitochondrial dysfunction is associated with reduced or partial fatty acid oxidation, which can lead to activation of stress kinases that can inhibit insulin signaling [69, 70]. Skeletal muscle of OVX mice shows lower use of palmitoylcarnitine and glycerol-phosphate substrates, decreased PGC1*α* expression, reduced mitochondrial content, and increased compensatory extramitochondrial ATP synthesis during exercise, most of which could be reverted by E2 treatment [71]. Thus impaired lipid use with E2-deficiency in skeletal muscle may lead to accumulation of intramyocellular fat, which has been implicated in insulin resistance (reviewed in [72]). There is also evidence that expression of the adipokine, adiponectin, and its receptor, AR1, is induced by estrogen in conjunction with mitochondrial biogenesis [73] and that adiponectin positively influences insulin sensitivity.

## 6. Clinical Significance and Therapeutic Potential

Clearly, metabolic effects of E2 are undeniable and there is an eminent need of therapeutics to protect menopausal at risk women from MetS, diabetes, and their associated comorbidities. There is debate over the question as to whether we should treat a natural phenomenon of the body such as menopause. Combined with increased duration of postmenopausal life, reduced exercise, a surfeit of food availability, and increasingly unhealthy food habits, E2-deficiency makes women more prone to MetS and its associated complications. However, treating E2-deficiency is not as easy as simply replacing E2 in menopausal women, since E2 and ER agonists are linked with aggressive cancers. Furthermore the therapeutic index is narrow since supraphysiological levels of E2 are as detrimental as E2-deficiency, and thus a targeted treatment strategy would be necessary. E2 compounds directed specifically to act in metabolic tissues such as skeletal muscle, liver, heart, adipose tissue, and pancreas with sparing of tissues linked with E2-sensitive cancers such as the ovaries, uteri, and breasts are considered potential viable treatments. Tissue selective E2 complexes (TSECs) are a combination of the selective receptor modulator bazedoxifene with conjugated E2 and have been shown to provide tissue-specific benefits of E2 such as reducing hot flashes, vulvar-vaginal atrophy, and menopausal osteoporosis in women [74] and improved CVD risk and MetS while surpassing the endometrium and breast in animal models [75, 76]. TSEC removed the requirement of progestin to protect the uteri and breasts, which has been contraindicative in WHI trials as being responsible for some of the results of increased CVD risk. While TSECs are still under clinical trials for use for MetS, newer, more innovative, efficient, and tissue-specific E2 receptor agonists are being investigated. For instance, E2 conjugated preparation with glucagon-like peptide-1 (GLP-1) resulted in superior efficacy over either hormone alone to reverse obesity, hyperglycemia, and dyslipidemia in mice and prevented reproductive endocrine toxicity and oncogenicity [77]. Such therapeutics holds the promise of relieving menopausal women from their E2-deficiency symptoms and preventing debilitating MetS. Even then, treatment with E2 and its conjugated versions will have to be administered in the form of personalized treatments especially for risky underlying conditions like preexisting tumors, type 1 diabetes, and preexisting heart conditions. Thus, some of the pressing questions remain: which patient population can safely benefit from E2 therapy, what new agents of HRT can be used to optimize the benefits and eliminate the risks of E2 therapy, and will there be ER*α*-specific, tissue-specific treatments and can these be used as a preventive in younger women or as treatment in older women with established CVD?

## 7. Conclusion

Taken together, the effect of E2 on diabetes is a combination of many factors, including direct effects on insulin signaling in insulin-sensitive tissue, effects on pancreatic beta cells regulating insulin release, its role in adipose tissue metabolism and energy expenditure, its effects in hepatic glucose production and on the hypothalamus to regulate food intake, and its effects on energetics and metabolism. It is very clear that E2 has tremendous potential as a therapeutic against diabetes and its associated complications, but it has to be administered in a safer form and personalized to individual needs.
